# Investigating the Effect of Polyetheretherketone (PEEK) Veneers on Bond Strength and Discoloration When Repairing Various Composites

**DOI:** 10.7759/cureus.73926

**Published:** 2024-11-18

**Authors:** Emel Arslan, Hatice Sevmez

**Affiliations:** 1 Department of Prosthodontics, Faculty of Dentistry, Bolu Abant Izzet Baysal University, Bolu, TUR

**Keywords:** bond strength, color change, composite, fiber composite, nanoceramic, peek, thickness

## Abstract

Objectives: This study aimed to examine the impact of the strength and color change of composite materials that could be utilized in the repair of polyetheretherketone (PEEK) veneer fractures.

Methods: The 220 nanoceramic-filled PEEK specimens used in the study were divided into four groups, and color measurements were made on a gray background (n=55): Group N, 1-mm-thick monochromatic composite; Group NN, 2-mm-thick monochromatic composite; Group F, 1-mm-thick 2-mm-diameter short fiber-reinforced composite, placed in the center and polymerized; and Group FF, 2-mm-thick 2-mm-diameter short fiber-reinforced composite, placed in the center and polymerized. The remaining mating surfaces were restored by filling with monochromatic composite and adhesion strength testing by re-measuring the color.

Results: The results of the two-way ANOVA indicated that there was a significant color change caused by both thickness and composite type (p<0.001). Additionally, a significant difference in bond strength was observed between the groups.

Conclusion: In PEEK restorations, short fiber-reinforced composites significantly increase bond strength, while the use of monochromatic composites of a certain thickness ensures that color change remains within acceptable limits.

Significance: Although PEEK has a superior mechanical structure, it does not meet the aesthetic expectations and needs to be veneered. However, fractures are frequently seen due to low bond strength. Therefore, this study is important because it aims to provide guidance to clinicians on aesthetic and mechanical methods that can be used in PEEK prosthesis repair.

## Introduction

Polyetheretherketone (PEEK) is a member of the polyaryletherketone (PAEK) family, known for its excellent chemical resistance, toughness, strength, heat resistance, and processability. Its phenylene rings are inert. The ether group provides flexibility and the ketone group provides rigidity. As a metal-free restoration option that can be used in dentistry, PEEK is increasingly important in prosthodontics and oral implantology because of its superior qualities [1]. Due to its excellent mechanical and aesthetic properties, it is suitable for supporting fixed and removable dentures and orthodontic wires [2]. PEEK can be combined with carbon fiber or glass and nanomaterials to enhance its properties. PEEK achieves a stress distribution comparable to that of natural teeth due to its shock-absorbing effect during occlusion and mastication [2].

By causing poor adhesion to resin cement, the low surface energy of PEEK is a significant drawback in prosthetic dentistry. In order to increase the surface energy of PEEK, conventional grinding, acid roughening, plasma spraying, and laser roughening techniques can be used. Some researchers have suggested that veneer coating may be necessary due to the lack of sufficient clarity of PEEK. PEEK can be used as a substructure material instead of metal alloys and zirconia due to its color and mechanical properties. The purpose of the veneer coating process is to improve the appearance of the material [3].

Dental restorations that have failed can be restored using one of three methods: replacement, repair, or restoration. The least intrusive of these therapies is restoration. A repair is a less invasive treatment than a replacement. When a restoration needs to have a damaged portion restored, repair is a less invasive process than replacement, which involves removing the entire restoration [4].

Mechanical or chemical processing alone is usually not sufficient for restoring the original strength. However, if used in conjunction, they can improve the repair performance [4]. Recently, it has also been observed that a bonding agent used as an intermediary layer can aid in the repair bond [5].

In recent years, manufacturers have concentrated on streamlining direct techniques by proposing bulk filler materials. One such material is a short fiber-reinforced composite. This material was first introduced in 2013 for highly stressed bearing areas (everX Posterior, GC, Leuven, Belgium) [6]. Its manufacturer offers everX Flow, a fluid variation of everX Posterior that exhibits superior fracture toughness (2.8 MPa/mm^2^) and a lower flexural modulus (9.0 GPa) compared to everX Posterior. However, its shrinkage stress remains higher than that of everX Posterior [7].

The primary objective of anterior restoration is to achieve optimal color harmony with the tooth and surrounding tissues. To this end, monochromatic resin composites have been developed to eliminate the need for shade selection [8]. These composites lack the inclusion of dyes or pigments, and their color-matching ability is contingent upon the distinctive structural composition of the material, which reflects the color of the surrounding dental structures [9]. Restorative materials are frequently subjected to dynamic challenges within the oral cavity, including masticatory forces, pH changes, and temperature fluctuations [10].

A significant amount of effort has been devoted to enhancing the aesthetics of the PEEK material in order to enhance its connectivity. Nevertheless, fiber composites were not implemented to fortify the framework. The aesthetic enhancement of the monochromatic composite has not been investigated in terms of its color change. The objective of this investigation is to rectify this deficiency in the literature. This study was designed to determine whether monochromatic resin composites could mask the opaque nature of PEEK and how short fiber-reinforced composites affect the bonding of PEEK restorations. 

The null hypothesis of the study was that short fiber-reinforced composites would have no effect on the bond strength of PEEK materials, while the alternative hypothesis was theorized that monochromatic composites would change the color of opaque PEEK.

## Materials and methods

Preparation of specimens

PEEK reinforced with 20% nanoceramic filler (BioHPP, Bredent GmbH & Co.KG, Senden, Germany) was cut in 5 × 5 mm dimensions and 2 mm thickness. Subsequently, all PEEK specimens were subjected to ultrasonic cleaning in distilled water for a duration of 60 seconds. Following this, the specimens were aged in distilled water at a temperature of 37°C for a period of 24 hours.

Measurement of color parameters of PEEK

Samples were analyzed in vitro using a spectrophotometer (VITA Easyshade®, VITA Zahnfabrik, Bad Säckingen, Germany) on a gray background. Prior to the application of the composite material, the L_0_, a_0_, and b_0_ values were measured. This was done in accordance with the manufacturer's instructions. The L, a, and b values were then measured again on the same gray background. Each sample was measured three times and averaged. The spectrophotometer was carefully calibrated during the measurements. To ensure standardization in the measurements taken after composite application, the first color measurement was performed in the same environment and at the same time.

Adhesion application 

The Visio.link adhesive system (Bredent GmbH & Co.KG, Senden, Germany) was applied to the connection surfaces of the samples using a microbrush for five seconds. The samples were then polymerized for 90 seconds using a Bre.Lux Power Unit (Bredent GmbH & Co.KG, Senden, Germany) with a power of 220 mW/cm^2^ and a wavelength of 370-400 nm, as recommended by the manufacturer.

Composite resin application

**Figure 1:**
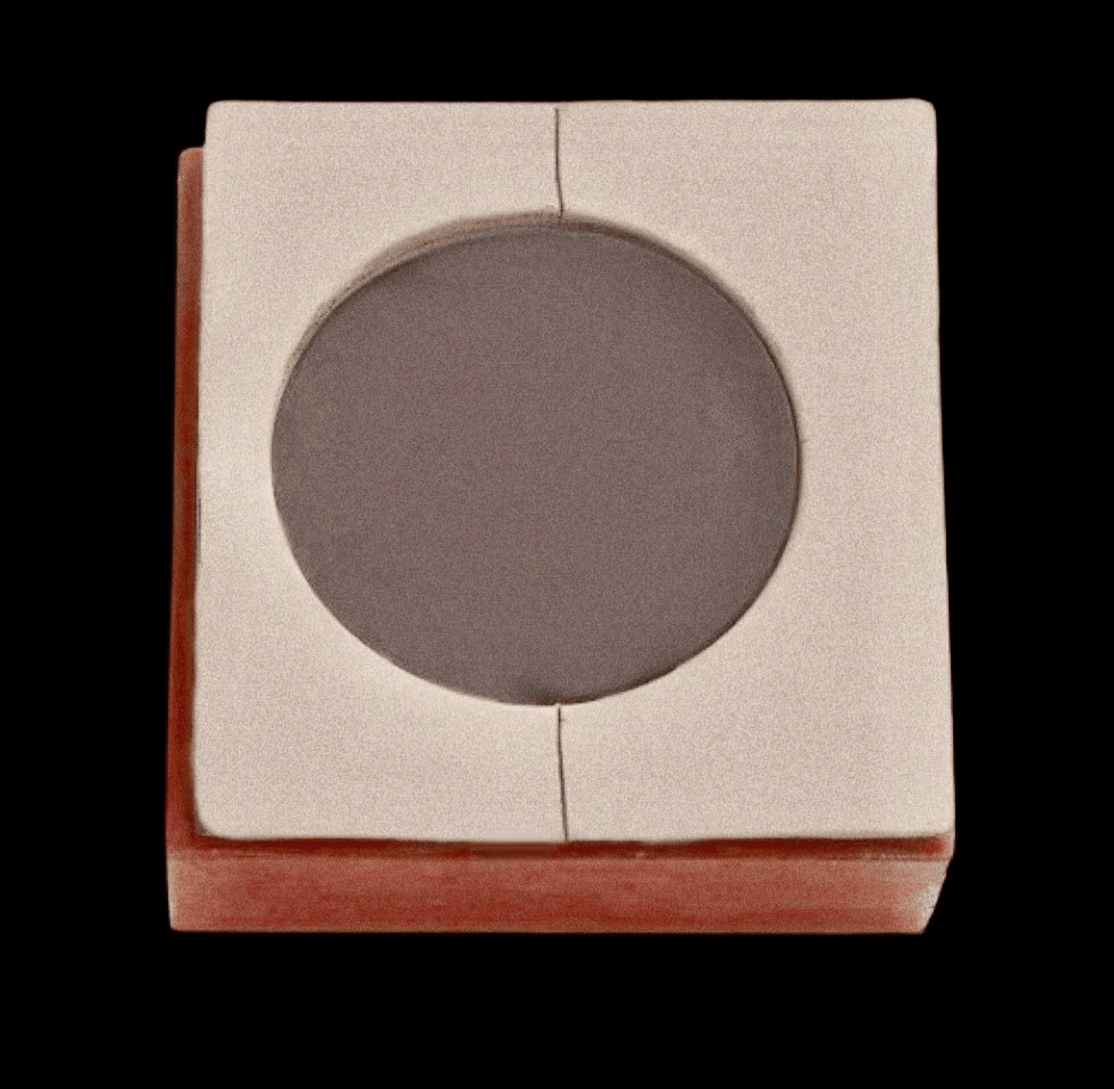
Special mold prepared for applying fiber composite on PEEK surface PEEK: polyetheretherketone

A total of four groups were randomly established based on the initial sample. For Group N, a 1-mm-thick layer of composite resin was applied to the surface of the specimens where the connection was to be made. Special square molds measuring 5 × 5 × 1 mm were used to standardize the composite resin (Neo Spectra ST composite; Kuraray, Osaka, Japan) to be applied to the specimen surfaces and to prevent overflow from the joint interface. The composite material was polymerized with the polymerization device of the adhesive system (Bre.Lux Power Unit) for 40 seconds. For Group NN, a 2-mm-thick layer of composite resin (Neo Spectra ST composite) was applied to the bonding surface of the specimens using specialized square molds with dimensions of 5 × 5 × 2 mm. The polymerization device of the adhesive system (Bre.Lux Power Unit) polymerized the composite material for 40 seconds. For Group F, a 2-mm-diameter layer of short fiber-reinforced composite (everX Flow, GC, Leuven, Belgium) was applied to the center of the joint surface using a specially prepared mold (Figure 1). It was then polymerized for 20 seconds, and the mold was then removed. The remaining areas of the PEEK surface were filled with composite (Neo Spectra ST composite). For Group FF, a 2-mm-diameter layer of short fiber-reinforced composite (everX Flow) was applied to the middle of the PEEK specimens using a special mold. The short fiber-reinforced composite was polymerized for 20 seconds. The remaining areas of the PEEK samples were filled with composite (Neo Spectra ST composite) and polymerized. The top surface of the PEEK samples was filled with 1 mm of composite (Neo Spectra ST composite E1) and polymerized for 20 seconds. The specimens underwent an aging procedure through thermocycling (Gökçeler Machine, Sivas, Turkey) for 10,000 cycles at 5-55°C, with a dwell time of 30 seconds.

Bond strength measurements

In order to perform the shear bond strength (SBS) test in a standardized manner, special molds with dimensions of 30 × 15 × 5 mm were prepared. Each mold contained a specimen embedded in self-curing acrylic resin (Integra, Istanbul, Turkey) at each end. SBS tests were conducted to assess the bond strength between the substrate and adhesive cement and also between the substrate and the PEEK-based substrates. The tests were conducted on a universal testing machine (Instron 8874, Norwood, MA, USA), which has a load cell of 25 kN capacity and a crosshead speed of 0.5 mm/s. In order to conduct the SBS tests, a special apparatus was constructed comprising two sliding parts. The SBS (MPa) was determined by dividing the breaking load (N) by the cross-sectional area of the specimen (mm^2^). 

Power analysis

Power and sample size analyses were conducted using G*Power (http://www.psychologie.hhu.de) (Ver. 3.1.9.2, Heinrich-Heine-Universität Düsseldorf, Düsseldorf, Germany) for the "repeated measures, within-between interaction." The study required 212 samples to detect a mean effect of f = 0.25 with an alpha of 0.05, 95% power, two measurements per group, a correlation of 0.5 between measures, and a correction for nonsphericity (ε) of 1. Therefore, it was decided to prepare 55 specimens per composite group.

Statistical analysis

The statistical analyses were conducted using IBM SPSS Statistics for Windows, V. 14.0 (IBM Corp., Armonk, NY, USA). To assess the homogeneity of composite and thickness variance distributions for each group (n = 55), the Kolmogorov-Smirnov test was applied, and normal distributions were found. The data composite and thickness measurements were subjected to a two-way ANOVA test, and the resulting values were subsequently compared using Tukey's test. The significance level of the p-value was p < 0.05.

## Results

A significant difference in ∆E00 and bond strength as affected by composite type and thickness was observed through a two-way ANOVA test (Table 1 and Table 2).

**Table 1:** Two-way ANOVA test for the effect of composite types and thickness on ∆E00 values *Tests of within-subjects effects Huynh-Feldt significant values. It was determined that the sphericity assumption was not met, p < 0.01, and Greenhouse-Geisser adjustment was higher than 0.75; therefore, Huynh-Feldt values were considered.

∆E00	Type III sum of squares	df	Mean square	F	Sig.*	Partial eta squared
Thickness	30.945	1	30.945	108.540	<0.001	0.334
Composite	308.716	1	308.716	1082.824	<0.001	0.834
Thickness × composite	37.633	1	37.633	131.997	<0.001	0.379
Error	61.582	216	0.285			
Total	6375.930	220				

**Table 2:** Two-way ANOVA test for the effect of composite types and thickness on bond strength values *Tests of within-subjects effects Huynh-Feldt significant values. It was determined that the sphericity assumption was not met, p < 0.01, and Greenhouse-Geisser adjustment was higher than 0.75; therefore, Huynh-Feldt values were considered.

Bond strength	Type III sum of squares	df	Mean square	F	Sig.*	Partial eta squared
Thickness	367.879	1	367.879	434.111	<0.001	0.668
Composite	2568.559	1	2568.559	3030.996	<0.001	0.933
Thickness × composite	125.668	1	125.668	148.293	<0.001	0.407
Error	183.045	216	0.847			
Total	128570.387	220				

∆E00 color change

**Figure 2:**
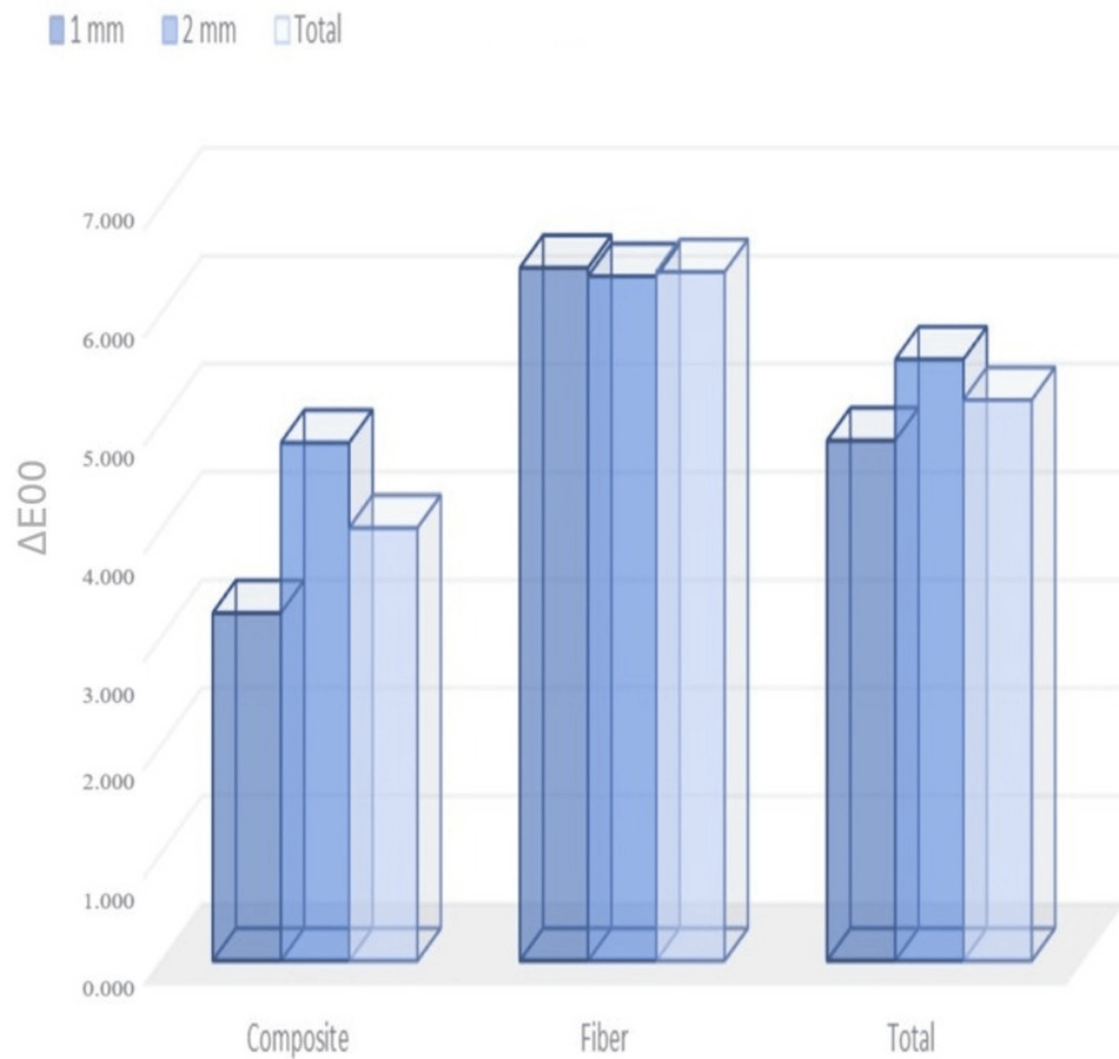
Mean and standard deviation of color change values according to material and thickness

The effect of color change on composite and thickness is shown in Table 1, where the main effect of composite on color change was significant (p < 0.001). The monochromatic composite's mean color change value was found to be 4.01 ± 1.01, while the composites with fiber had a color change of 6.38 ± 0.41. The main effect of applied thickness was also statistically significant (p < 0.001) (Figure 2). The color change was 3.22 ± 0.05 when applied at 1-mm-thick composite and 4.80 ± 0.90 when applied at 2 mm. There were significant interactions between thickness and composite (p < 0.001). The samples with a composite thickness of 1 mm showed the lowest color change. The groups where the fiber composite was applied did not show a significant difference in thickness but did show the highest color change. Additional results of multiple color change comparisons can be found in Table 3.

**Table 3:** ∆E00 color change descriptive statistics a*, b: no difference between composite with the same letter. A, B, C: no difference between composite and thickness interactions with the same letter. ηp^2!^: partial eta squared

∆E00	1 mm (n = 110)	2 mm (n = 110)	Total	η_p_^2!^
Monochromatic composite (n = 55)	3.22 ± 0.05^A^	4.80 ± 0.90^B^	4.01 ± 1.01^a^*	0.930
Fiber composite (n = 55)	6.42 ± 0.52^C^	6.34 ± 0.26^C^	6.38 ± 0.41^b^	0.350
Total	4.82 ± 1.65	5.57 ± 1.02	5.19 ± 1.42	0.291

Bond strength results

**Figure 3:**
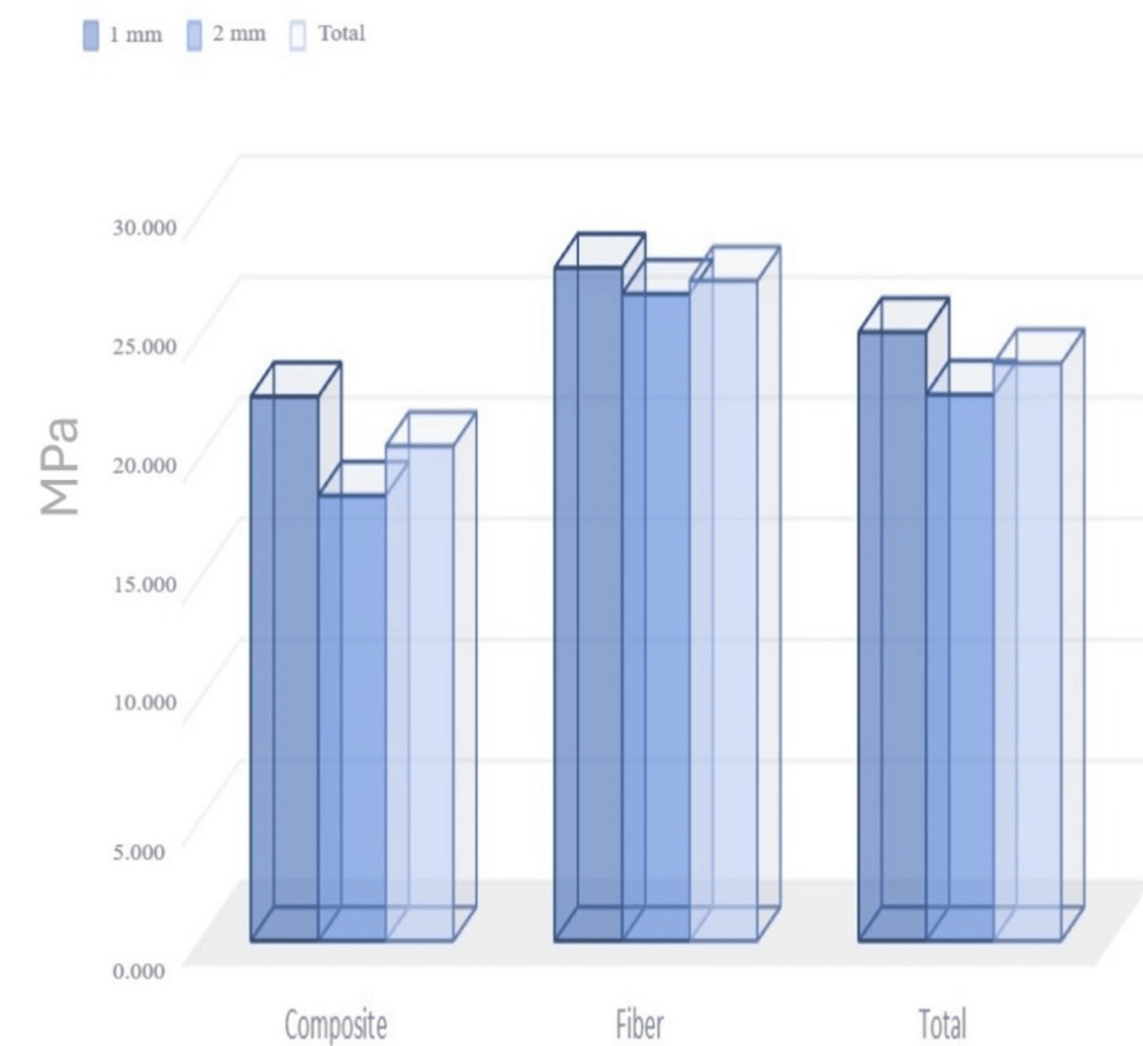
Mean and standard deviation of bond strength values according to material and thickness

Table 1 shows the effect of bond strength on composite and thickness. The main effect of composite and thickness on bond strength was found to be significant (p < 0.001) (Figure 3). The average bond strength value for monochromatic composite was 20.45 ± 2.27, while for fiber-applied composites, it was 27.28 ± 1.03. The lowest bond strength value was 18.4 ± 0.77 in the 1-mm-thick composite group, and the highest value was 27.82 ± 0.82 in the 1-mm-thick fiber-applied group. Table 4 provides comparisons of multiple other changes.

**Table 4:** Descriptive statistics on bond strength a*, b: no difference between composites with the same letter. A, B, C, D: no difference between composite and thickness interactions with the same letter. ηp^2!^: partial eta squared

Bond strength	1 mm (n = 110)	2 mm (n = 110)	Total	η_p_^2!^
Monochromatic composite (n = 55)	22.5 ± 1.12^A^	18.4 ± 0.77^B^	20.45 ± 2.27^a^*	0.930
Fiber composite (n = 55)	27.82 ± 0.82^C^	26.75 ± 0.93^D^	27.28 ± 1.03^b^	0.350
Total	25.16 ± 2.85	22.57 ± 4.28	23.87 ± 3.85	0.291

## Discussion

The study's primary hypothesis, which stated that the materials would have no effect on bond strength and color change, was rejected. However, H1A, which hypothesized that monochromatic composites would change the color of PEEK, and H1B, which hypothesized that samples containing short fiber-reinforced composites would have higher bond strength, were both accepted.

Previous research has indicated that the SBS values between PEEK and composite resin are higher when primers containing methyl methacrylate (MMA) are used. Visio.link, a primer combining MMA, dimethacrylates, and pentaerythritol triacrylate (PETIA), was also used in the current study [11-14]. While MMA may result in swelling and make it easier for dimethacrylate monomers to adhere to the composite resin through methacrylate groups, PETIA could assist in the disintegration of the PEEK surface [14]. An acceptable SBS for medical purposes is 10 MPa or higher, according to Behr et al. [15]. Barto et al. [16] noted that an adhesive bond of 10 MPa was required to withstand shear for a typical bonding interfacial area of 20 mm^2^ and an average anterior occlusal force of 200 N. Moreover, it has been documented that a primer by itself is unable to produce a clinically meaningful bond strength. In the present study, the bonding strength between PEEK and the composite was measured using a short fiber-reinforced composite.

Consistent with the results of Lassila et al. [17] and Garoushi et al. [18], this study revealed that the short fiber-reinforced flowable composite (everX Flow) had much higher bond strength values. These investigations confirmed the short fiber-reinforced flowable composite's excellent flexural qualities and fracture toughness. While this dentin substitute material's fracture toughness was comparable to that of dentin, fewer catastrophic failures and a fracture behavior more like that of nature were seen. It is significant to remember that the critical fiber length (lc), or the smallest length of high aspect ratio fiber fillers that can successfully reinforce the resin composite, is also influenced by the fibers' adherence to the polymer matrix. By strengthening the material's resistance to crack propagation and lowering the stress intensity at the crack tip, the addition of short fibers stops cracks from spreading [7]. On the other hand, when doing semi-(in)direct computer-aided design (CAD)/computer-aided manufacturing (CAM) restorations, everX Flow is more suited as a liner in modest quantities, particularly above the dentin sealant, to rectify geometry and preserve the dentin connection. In the present study, findings showed that composites with 1-mm-thick fiber reinforcement had a stronger bond. Furthermore, the short glass-fiber composite that was employed as the base in an in vitro investigation by Eapen et al. [19] exhibited enhanced fracture toughness compared to the negative control group after being coated with a nanohybrid composite layer that was only 1 mm thick. The nanohybrid composite groups in this study produced a lower bonding strength. Because of the aforementioned drawback, direct composite restorations might not be the ideal option when PEEK veneer fractures.

One major contributing cause of the cosmetic failure of restorations is the yellowing of composite materials, especially at the edges. Restorations in aesthetic areas may frequently need to be replaced as a result [20]. Resin composites have been noted to have the ability to absorb liquids, including water, which may cause discoloration [21]. Resin composite filler particles can absorb water on their surfaces, but they do not absorb water into the substance itself. As a result, composites with a larger percentage of resin matrix absorb more water and have a weaker link between the resin matrix and filler particles. As a result, there may be interfacial gaps created between the resin matrix and filler, and the swelling and plasticizing processes may cause microcracks to form inside the resin matrix. These elements make it easier for stains to seep through and for the restorations to discolor [22]. The analysis of the study showed that the samples' colors had changed.

Discoloration assessment can be carried out using visual or instrumental methods. Spectrophotometry was used in our study to exclude the possibility of subjective interpretation in comparisons of ocular colors. In dental materials research, spectrophotometry is a tried-and-true method [21]. Numerous investigations have demonstrated that color variations are perceptible to the human eye when the ∆E values fall between 1 and 3. Clinically inappropriate values are those that are higher than 3.3 [23,24]. The study findings demonstrate that all groups' discolorations were more than the clinically acceptable threshold, with the exception of the Neo Spectra composite 1 mm group. When PEEK specimens are repaired with short fiber-reinforced composites, discoloration may result, particularly in the anterior area, and this may cause the restoration to fail. 

Considering that triethylene glycol dimethacrylate (TEGDMA) is a component of the short fiber composite, it may have a higher inclination to release monomers into aquatic environments [25]. Determining the exact effect of dental formulations on color stability is a difficult task. The degree of monomer conversion is strongly associated with the amount of unreacted monomers present. Reduced unreacted monomer concentration, reduced water absorption, and enhanced color stability are the outcomes of a higher degree of monomer conversion. The manufacturer's instructions state that the color stability of the short fiber-reinforced composite is equivalent to other evaluated bulk-fill and traditional resin composites, despite the fact that it is eventually supposed to be covered with a more aesthetically pleasing composite [26].

The degree to which the resin composite and tooth are shade-matched can be influenced by a variety of factors, including the size and proportion of the filler, the makeup of the matrix, the size of the restoration, the way the composite is layered, and the composite's color and brand [27]. It has been shown that in comparison to resin composites with bigger filler particles, those with smaller, irregularly shaped filler particles allow improved light transmission [28]. It has also been noticed that in comparison to composites with spherical filler particles, irregularly shaped filler particles increased b* values and decreased a* values. ΔE00 values <1.8 were not yielded in another trial, which is regarded as clinically appropriate [29]. The findings of the study were also found to be compatible with these studies.

One particular problem in composite repair is the surface of aged composites, which often lacks unreacted double bonds that may be bonded to the new composite. Some studies have used 10,000 cycles, which is equivalent to a year of physiological aging in the oral cavity, and this should be sufficient to promote a significant increase in composite double bond conversion, even though there is disagreement regarding the standard laboratory aging methods that replicate this effect [30].

This in vitro investigation to assess bond strength and color stability in the repair of PEEK material has its own methodological limitations. The aim of this study was to model the long-term effects of thermal cycling and predict the clinical performance of composites. Factors in the oral cavity, such as hot and cold beverages, saliva, and the chewing process, can significantly alter the color change and bond strength. Furthermore, the PEEK material used, composites, and methods tested can be varied. This study will guide future in vitro and in vivo studies that can give clinicians an idea in terms of repair.

## Conclusions

Within the limitations of the research, the following conclusions can be made: The thickness and type of composite utilized have a significant impact on color change and bond strength. Short fiber-reinforced composites can be employed for repair in posterior areas with high masticatory force, as they enhance the bond strength with PEEK. Monochromatic composites are preferable for PEEK restoration repair in the anterior region, as they exhibit an acceptable color change in the anterior region, where aesthetics are of paramount importance.
